# Topical Atropine in the Control of Myopia

**Published:** 2016

**Authors:** Virgilio GALVIS, Alejandro TELLO, M. Margarita PARRA, Jesus MERAYO-LLOVES, Jaime LARREA, Carlos JULIAN RODRIGUEZ, Paul Anthony CAMACHO

**Affiliations:** 1 Centro Oftalmologico Virgilio Galvis, Floridablanca, Colombia; 2 Faculty of Health Sciences, Universidad Autonoma de Bucaramanga (UNAB), Floridablanca, Colombia; 3 Instituto Universitario Fernandez-Vega Oviedo, Spain; 4 Fundacion Oftalmológica de Santander FOSCAL, Floridablanca, Colombia

**Keywords:** Topical Atropine, Myopia, Progression

## Abstract

Atropine has been used for more than a century to arrest myopia progression. Compelling evidence of its protective effect has been reported in well-designed clinical studies, mainly performed during the last two decades. However, its exact mechanism of action has not been determined. Experimental findings have shown that the mechanism is not related to accommodation, as was thought for decades. A review of the published literature revealed a significant amount of evidence supporting its safety and efficacy at a concentration of 1.0%, and at lower concentrations (as low as 0.01%).

## INTRODUCION

During the preceding decades, a noteworthy increase in myopia prevalence has been reported in many countries, including in Southeast Asia. This increase, which has occurred over only 25 to 50 years, has focused renewed attention on the crucial effect of environmental factors and has prompted a growing interest in pharmacological treatments that can help stop the progression of myopia (1-7).

In 1611, Kepler proposed his hypothesis of near work as the cause of myopia, suggesting that reading and performing visual tasks at short distances in childhood accustomed the eye to near objects (8-10). Due to Kepler’s work, accommodation was linked to myopia. Several mechanisms related to accommodation and/or convergence were proposed during the next two centuries (11, 12). The good, well-documented results that were obtained using bilateral atropine treatments during the 20th century demonstrated that convergence likely has no effect on myopia onset or progression, as children receiving bilateral atropine continued to perform near-vision work, and therefore converge, but the severity of myopia did not increase (13-16). In addition, experimental evidence against accommodation as a primary determinant in the etiology of myopia has recently emerged, including the finding that experimental myopia could be induced in primates even after destruction of the ciliary ganglion or the Edinger-Westphal nucleus, which eliminated the accommodative reflex (17). Stone et al. (1991) reported that atropine attenuated the excessive axial elongation related to form deprivation in chicks, although they do not have muscarinic receptors in the ciliary body (18). McBrien et al. (1993) showed that it was possible to induce experimental myopia in small mammals (grey squirrels) that do not have a functional accommodative system (19). The same author in the same year also reported, in accordance with the findings of Stone et al., that atropine slowed the development of experimental myopia in chicks, indicating that a non-accommodative mechanism was involved in experimental myopia onset and progression, since ciliary muscle in chicks contracts through a nicotinic not a muscarinic mechanism (20).

Modern epidemiological studies have produced contradictory results concerning near work and myopia. Several studies have found that near work is related to a higher prevalence and degree of myopia (21-29); children who read uninterruptedly or at a closer distance have a higher probability of being myopic, and stabilization of myopia by the age of 15 years may potentially be associated with less time spent on near work activities (27, 30, 31). However, other findings have not supported a significant effect of near work on myopia (21, 29, 32-37). In the Orinda Longitudinal Study of Myopia, Jones et al. studied a group of children from California, U.S.A. and found that near work (reading, watching television, studying, using the computer, or playing video games) was not a significant risk factor in myopia onset (32). In the Singapore Cohort Study of the Risk Factors for Myopia (SCORM), Saw et al. thoroughly assessed near work (books per week, hours per day of reading, computer use, playing video games, and watching television) and also found that none of these variables was a significant risk factor for myopia (33, 34). Very recently, Zadnik et al. used data from the collaborative longitudinal evaluation of ethnicity and refractive error (CLEERE) Study in the United States (including Caucasian, African-American, Hispanic, and Asian children) and found that near work was not predictive of myopia onset, either in univariate or multivariate models (37).

## HERITABILITY VERSUS ENVIRONMENT IN MYOPIA

Heritability has been identified for more than a century as an influencing factor, and its link to myopia has been confirmed by many genetic and epidemiological studies during the last 50 years (7, 12, 38-46). However, as individuals from the same family frequently share common environmental conditions, heritability studies can reflect overestimations (7, 42, 43). The discovery of more than 40 genetic loci related to the development of myopia has supported the existence of a genetic contribution to this condition (1, 41, 42, 47, 48); one important predictor for the onset of myopia is a parental history of myopia (21, 37, 43, 49-55). In the SCORM study, Saw et al. found that schoolchildren with two myopic parents had an increased risk of myopia (1.6 times) compared to children with no myopic parent (32, 34, 42, 43, 49). In addition, according to the recently reported results of the Growing Up in Singapore towards healthy outcomes (GUSTO) study, Chua et al. suggested that genetic factors may have a larger impact on the early development of refractive error than environmental factors. In multivariable regression models, 3-year-old children with two myopic parents were more likely to have a more myopic spherical equivalent and longer axial length, and to be more likely to have myopia, than children whose parents were not myopic (56). However, other researchers explain that while this relationship is compatible with the idea of a genetic basis for myopia, it does not establish a definitive relationship because, as previously mentioned, parents and their children also share common environmental factors (7). In addition, the influence of parental myopia on the refractive error of schoolchildren and teenagers has not been a universal finding. In 1886, Cohn found that only 2.7% of 1,004 myopic schoolchildren reported that one of their parents was myopic or possibly myopic (12). Quek et al. (2004) reported no statistical difference in myopia incidence in students aged 15-19 years according to parental history (57). However, both studies, performed more than a century apart, had the limitation that the parental refractive status was established by questioning the children and teenagers rather than the parents (12, 57). The interaction between heredity and environment has been a subject of controversy for long time (12, 44, 45). In a three-generation study of children from Hong Kong and Northern China, Wu and Edwards found that the influence of family history (at least one myopic parent) on the risk of having myopia was higher in the second generation (parents’ generation), than in the third generation (children’s generation). This finding supports a stronger effect of environmental factors than heritability on the development of myopia (7, 54).

Pioneering studies by Wiesel and Raviola in the late 1970s and later studies by Wallman and coworkers confirmed that deprivation of visual stimulus caused myopia in young animals in multiple species (58, 59). Those experiments demonstrated the effect of environment on the refractive status of the eye in animals, but this does not necessarily indicate that human myopia is largely environmental (2, 58-60). Current evidence, including experimental studies, seems to support the premise that juvenile myopia development is driven by both genetic and environmental factors (34, 43, 51, 61-64). However, the mechanism by which genes identified as responsible for experimental myopia determine the appearance of that refractive error in humans has not yet been determined (51, 61, 65). Mutti et al. (1996) discussed the nature versus nurture debate as each affects the development of myopia. Traditionally, it was assumed that one or the other affected the development of myopia. Now, it appears that both do, and research is being conducted to examine the extent of the effect each one has (66). Morgan and Rose (2005) reported that high heritability does not indicate an established limit to possible changes generated by environmental factors. They indicated that the concept that populations of East Asian origin have a genetically determined higher risk of myopia was not supported by the low prevalence reported in populations in non-urban areas of several countries in the region (Mainland China, Taiwan, Japan, and Singapore), or by the high rate of myopia found among other ethnic groups (including Indians) with different genetic information migrating to Southeast Asia (7, 67-70). Examples of low prevalence of myopia in East Asian people include the report by Chang et al., who cited a study by Chen, performed in the 1980s, that found only a 9.7% prevalence of myopia in non-aboriginal students in Taiwan (69). Lin et al. reported a rate of myopia of 20% among aboriginal schoolchildren (68). More recent findings in China and Singapore also seem to support that East Asian people do not have a significantly higher genetic predisposition for myopia than other ethnic groups, as low prevalence estimates have been reported in some ethnic groups from those countries (71-74). In addition, the premise that an East Asian genetic background may increase susceptibility to risk factors does not seem to be valid considering the recently reported findings of a significant increase in myopia in young adults of Indian origin living in Singapore over a period of 13 years (1996-1997 and 2009-2010) (3). This increase in prevalence in Indian individuals was higher than in individuals of Chinese and Malay ethnic groups of the same age (3). However, myopia in India is much less prevalent (75). These findings suggest that environmental conditions, including intensive education and limited time spent outdoors, are directly related to the high prevalence of myopia in all ethnic groups of Singapore, indicating that genetic influence may not be very important (7, 67, 68, 74). A propensity to develop myopia in so-called ‘‘myopigenic’’ milieus seems to be a feature common to most humans (7, 66, 74). Detailed reviews of the research into the genetics of myopia have been published elsewhere by different authors (47, 51, 61, 76-79).

## ATROPINE USE IN THE CONTROL OF MYOPIA

By the mid-1800s, atropine was frequently used in ophthalmology for pupillary dilation to examine the posterior segment of the eye and as a temporary treatment to improve vision in cases of cataracts. It was also used to induce mydriasis during cataract surgery and to prevent or break the posterior synechia in cases of uveitis. At that time, it was not used in myopia treatment (80-82).

Donders (1864) was the first to recommend atropine as a treatment for myopia when he suggested it for suspected spasms of accommodation in myopic patients (11). One hundred years ago, Pollock was the first to employ prolonged use of atropine for the treatment of myopia (for a duration of several months to almost a year); the therapy also required affected children to avoid reading and writing (13, 83, 84). However, in the following decades of the 20th century, pharmacological treatment of myopia was not pursued. Few researchers from the 1930s to the 1990s conducted new studies (85-97). As previously mentioned, several of those studies completely disproved the hypothesis of convergence as the main cause of myopia, as children in those studies continued to read with both eyes, and therefore converge, with no signs of worsening myopia. In spite the evidence of the effectiveness of atropine treatment, it was not popular among ophthalmologists and had notable detractors (98-101).

A compelling body of evidence has recently emerged from several well-designed studies, including large groups of children, mainly from Asia. Shih et al. (1999) studied the daily use of different concentrations of atropine in 186 schoolchildren followed for up to two years (102). They found that the mean progression of myopia was 0.04 ± 0.63 diopter (D) per year in the group receiving a solution of 0.5% atropine; 0.45 ± 0.55 D per year in the group that received 0.25% atropine; 0.47 ± 0.91 D per year in the 0.1% atropine group, and 1.06 ± 0.61 D per year in the control group who received 0.5% tropicamide. They concluded that although all three concentrations of atropine had a significant protective effect with regard to slowing myopia progression, 0.5% atropine was the most effective (102). In 2001, the same researchers reported the results of another clinical trial that evaluated the effects of atropine with multifocal lenses to decrease the progression rate of myopia in 188 children assigned to three treatment groups. The first group was treated with daily 0.5% atropine concomitantly with use of multi-focal eyeglasses, the second group used only multi-focal eyeglasses, and the third group used single vision eyeglasses. The study had a follow-up time of at least 18 months. The researchers found that over 18 months, the mean progression of myopia in the group treated with atropine and multi-focal eyeglasses (0.42 D) was significantly less than the multi-focal (1.19 D) and single-vision groups (1.40 D) (103). The study Atropine in the Treatment of Myopia (ATOM 1) by Chua et al. (2006) was a randomized, double-blind, placebo-controlled trial including 400 children. It showed that 1.0% atropine eye drops applied daily in one eye over a period of 24 months reduced the progression of myopia by 77% compared with the untreated eye (1.2 D in the control group compared to 0.28 D in eyes treated with atropine) (104). The primary effect of atropine appeared to be by slowing the growth of vitreous chamber depth, which in turn decelerate axial length increase (105).

Both concentration and frequency of atropine have been modified to minimize the side effects while trying to maintain the benefits. Chou et al. (1997) proposed that application of 0.5% atropine eye drops once per day was effective for slowing the progression of refractive error, even in children with severe myopia (106). As mentioned earlier, this group of researchers had also compared different concentrations of atropine and concluded that although 0.5% atropine was the most effective, the dropout rate may have reduced its effectiveness. Therefore, in 1999 it was suggested that because daily drops of 0.1% and 0.25% atropine were well-tolerated, those concentrations could be used initially to control the progression of myopia in children with rapid progression or in those who tended to have severe or early-onset myopia (102). More than a decade later, two additional studies that used low-concentration atropine and included medium-term follow-up were published. Wu et al. (2011) studied a group of 117 children (97 children received 0.05% or 0.1% atropine, 20 children were included as a control group). The mean follow-up length was 4.5 years. They used for analysis a model containing both fixed and random effects (mixed model) and found that the adjusted progression of myopia in the treated group was significantly lower than that of the control group (-0.23 D versus -0.86 D per year) (107). In 2012 the results of the Atropine for the Treatment of Myopia 2 (ATOM 2) study indicated that the efficacy of a very low concentration (0.01%) atropine applied once nightly did not significantly differ from that of 0.1% and 0.5% atropine solutions for control of myopia progression, and had minimal side effects (108). The ATOM 2 study reported that over two years, the mean progression was −0.30 D in the 0.5% group, −0.38 D in the 0.1% group, and −0.49 D in the 0.01% group. All groups had progression significantly lower than the control group reported in the ATOM 1 study, which was -1.20 D for the same two-year period. However, cessation of treatment often resulted in a myopic rebound effect, which was more pronounced in eyes that received 1.0%, 0.5% and 0.1% atropine than in eyes that received 0.01% atropine (109). Recently, researchers reported that younger age, greater severity of myopia, and faster progression were risk factors of progression for a subgroup of children despite receiving atropine treatment (110). The five-year results of the ATOM 2 study were published recently. After receiving atropine at different concentrations for 24 months (phase 1), individuals received no treatment for 12 months. Children who presented with myopia progression > 0.50 D were then restarted on 0.01% atropine therapy for a further 24 months. As 0.01% atropine showed the lowest rebound effect, it was the most effective concentration to reduce the progression of myopia at three and five years, with five-year overall change of -1.38 D, compared with -1.83 D in the 0.1% group and -1.98 D in the 0.5% group (111). However, when considering all children that received 0.1% atropine or 0.01% atropine compared to those who received 0.5%, atropine there were significant differences in progression during the first year; slower progression was observed in the 0.5% group. In terms of progression during the second year, when comparing all children who received 0.01% atropine versus those who received 0.5%, the difference was not significant, nor was significant in the same period among those who received 0.1% atropine versus those who received 0.5%. These results suggest that a promising approach would be for myopic children to use a more concentrated atropine solution (0.5%) during the first year and a lower concentration (0.01%) the second year (112).

In 2012, we suggested that applying one eye drop every week, in comparison with one per day, would facilitate compliance among young patients. Our study used 1.0% atropine once per week in conjunction with photochromatic progressive addition lenses (PAL) and ocular hypotensive eye drops, was well tolerated by the patients, and was very effective at stopping myopia progression (113). In a group of 33 patients (66 eyes) aged 6 to 16 years (mean, 11.9 years) treated with ocular hypotensive drops, the baseline spherical equivalent was -4.52 D. At the one-year follow-up, the spherical equivalent was -4.46 D (P = 0.015). This slight reduction of the magnitude of myopia was attributed to the hyperopic shift secondary to the cycloplegic effect of atropine. Progression was essentially zero (113).

As lower concentrations of atropine have been shown to be effective, and considering that the effect can last for up to 2 weeks, we suggest that a future study should investigate a course of treatment that uses 0.5% atropine therapy once or twice per week for one year, which is then changed to 0.01% atropine administered daily (112).

Modern studies outside Asia have also reported good outcomes. Polling et al. (2016) studied 77 myopic children of diverse ethnicity (European, Asian, and African) who received 0.5% atropine eye drops every day. Sixty children received the treatment for 12 months. The most common adverse events were photophobia (72%), reading difficulties (38%), and headache (22%). Myopia progression before treatment was −1.0 D/year ± 0.7, and drastically diminished during the treatment period to −0.1 D/year ± 0.7. Those children who stopped the therapy had a progression of −0.5 D/year ± 0.6 (114).

Several meta-analyses have been performed on this topic in the past six years. Walline et al. (2011) concluded that the most probable effective treatment to diminish myopia progression was anti-muscarinic eye drops. However, side effects and unavailability limited clinical use (115). Song et al. (2011) published a meta-analysis of six randomized clinical trials that analyzed the annual rate of myopia progression after daily atropine application over one year. They concluded that lower concentrations (0.05%, 0.1%, and 0.25%) of atropine were not effective because myopia may still progress during use. However, when higher concentrations were used (0.5% and 1%), the progression was controlled for between 6 and 24 months in the diverse studies (116). Another meta-analysis by Li et al. (2014) concluded that atropine could significantly slow myopia progression in children, but showed greater effects in Asian than in Caucasian children. The weighted mean differences in myopia progression between treated and control groups in cohort studies and clinical trials including Asian children were 0.54 D per year and 0.55 D per year, respectively. Progression was smaller (0.35 D per year) in cohort studies including Caucasian children (117). In 2016, Huang et al. published their network meta-analysis of interventions for myopia treatment. They included 30 clinical trials in the analysis (5422 eyes) and performed a random effects network meta-analysis combining direct and indirect evidence. When comparing mean annual change in refraction (diopters/year) and mean annual change in axial length (millimeters/year) with placebo or single-vision eyeglasses, they found that 1.0% and 0.5% atropine (refraction change: 0.68 D; axial length change: -0.21 mm); 0.1% atropine (refraction change: 0.53 D; axial length change: -0.21 mm) and 0.01% atropine (refraction change: 0.53 D; axial length change: -0.15 mm) markedly slowed myopia progression. However, on direct comparison, 1.0% and 0.5% atropine had slightly higher effects compared to 0.01% atropine (refraction change: 0.10 D; axial length change: -0.07 mm) (118).

A clear advantage of very low atropine concentration is tolerance. Several studies have shown that side effects of low-concentration atropine are very uncommon. In 2011 Wu et al. published the results of a retrospective, case-control study including 117 children who received 0.05% atropine, and if progression of more than -0.5 D during a 6-month follow-up was observed, were changed to 0.1% atropine and were followed for at least 3 years. No side effects were reported (107). The ATOM 2 study reported that upon restarting 0.01% atropine in children who showed progression after a 12-month atropine washout (n = 192), there was a mean increase in photopic pupil size of approximately 1 mm and a loss of accommodation of 2.00 to 3.00 D, which were similar to the changes observed when the children were initially assigned to 0.01% atropine during phase 1. These ocular side effects were considered clinically insignificant. Children were offered progressive addition or photochromatic (tinted) glasses if they had problems with near vision when using the single vision eyeglasses, or experienced glare. During phase 1, 7% of children receiving 0.01% atropine requested such glasses, but no child who had restarted 0.01% atropine requested glasses. Pupil size and accommodation returned to levels similar to those in untreated children when examined two months after cessation of 0.01% atropine (111). In 2013 Cooper et al. performed a phase I clinical trial including 12 children with brown irises (one eye included in the study group and the fellow eye used as control), and found that 0.02% atropine was the maximum concentration that could be administered daily without a clinical effect, having defined the target toxicity level as an accommodative amplitude below 5 D, a difference in pupillary diameter equal to or greater than 3 mm, and/or a failure to read very small print (J1) while wearing distance correction (119). Nishiyama et al. found that although accommodation decreased by a mean of 1.5 D and the pupil diameter increased in size by mean 0.7 mm, the subjective symptoms of a group of 16 children receiving 0.01% atropine eye drops daily were minimal after two weeks (120). Recently, Loughman and Flitcroft performed a tolerance study on 14 young Caucasian adults in Ireland, who received one drop of 0.01% atropine in both eyes every day for five days. Photopic pupil size increased between 1.08 and 1.31 mm and amplitude of accommodation decreased slightly; however, there were no negative effects on visual acuity or reading speed. Although there was a slight increase in glare, there was no significant negative impact on quality of life, which was associated with low-dose topical atropine (0.01%) (121).

The effectiveness of the lowest atropine concentration (0.01%) in the ATOM 2 study has been replicated outside Asia (111). A retrospective case-control study performed by Clark and Clark on 60 children (ethnically diverse in the United States) used 0.01% atropine and found diminished rates of myopic progression after one year (-0.1 D/year) compared to no medication (-0.6 D/year). However, despite receiving atropine, three patients experienced rapid myopic progression (122).

Additionally, low-concentration atropine has demonstrated some effect in the prevention of myopia onset in children who do not yet present it (pre-myopic). Atropine (0.025%) was administered to children aged 6 to 12 years with a spherical equivalent between +1.00 and -1.00 D, who were followed for at least 12 months. Twenty-one percent of the children receiving atropine became myopic, compared to 54% of those in a control group (123).

In summary, robust evidence supports atropine use to slow the progression of myopia. Some concerns regarding long-term safety have not yet been resolved. However, the general consensus of the clinical studies is that the treatment is safe. Lower concentrations (0.01%, 0.1%) are better tolerated and could be a very good option in clinical practice (111, 122, 124). However the weaker efficacy could be a concern (116). A once- or twice-a-week application could be an alternative treatment option for the use of high-concentration atropine (0.5% or 1.0%) (112, 113). 

Currently, the prescription of atropine eye drops in myopic children has not yet become standard practice in the Western hemisphere; it is commonly used in Asian countries such as Taiwan and Singapore, and has been recommended for more than 15 years by the Ophthalmological Society of Taiwan as a therapeutic alternative to slow myopia progression (124-126). Now may be the time to begin the use of this therapeutic alternative in daily clinical practice around the world.
